# 17 Ways to Say Yes: Toward Nuanced Tone of Voice in AAC and Speech Technology

**DOI:** 10.3109/07434618.2015.1037930

**Published:** 2015-05-12

**Authors:** Graham Pullin, Shannon Hennig

**Affiliations:** ^a^ Interaction Design, Duncan of Jordanstone College of Art and Design (DJCAD), University of Dundee, Dundee, UK; ^b^ Inclusive Communication, Wellington, New Zealand

**Keywords:** Expressiveness, Intonation, Speech-generating devices, Interaction design, Participatory design

## Abstract

People with complex communication needs who use speech-generating devices have very little expressive control over their tone of voice. Despite its importance in human interaction, the issue of tone of voice remains all but absent from AAC research and development however. In this paper, we describe three interdisciplinary projects, past, present and future: The critical design collection Six Speaking Chairs has provoked deeper discussion and inspired a social model of tone of voice; the speculative concept Speech Hedge illustrates challenges and opportunities in designing more expressive user interfaces; the pilot project Tonetable could enable participatory research and seed a research network around tone of voice. We speculate that more radical interactions might expand frontiers of AAC and disrupt speech technology as a whole.

## Introduction

As a person with amyotrophic lateral sclerosis (ALS) who used a speech-generating device (SGD) as part of communication, Colin Portnuff (2006) described his need for more expressive tone of voice: “I want to be able to sound sensitive or arrogant, assertive or humble, angry or happy, sarcastic or sincere, matter of fact or suggestive and sexy” (p. 6).

Tone of voice is a complex concept that goes beyond Bunnell and Pennington's (2010) definition of expressiveness as the ability “. . . to render the prosodic features of an utterance to reflect the different meanings talkers might want to convey” (p. 78). It is our position that tone of voice is a high level social construct that includes communication partners’ subjective impressions – perceptions that are further refined by a knowledge of how a person or group habitually speaks.

No matter that tone of voice, whether in spoken or synthesized speech, is difficult to define: it cannot be avoided. Even when an author annotates a line of dialogue as being delivered “. . . in a tone that was utterly meaningless” (Hammett, 1929, p. 16), this is meaningful in that it is remarkable. Alm and Newell (1996) considered the ways in which speech-generating devices may support but also undermine people with complex communication needs who use them as part of augmentative and alternative communication (AAC). They noted that these individuals face the challenge of being seen as constantly sending out inappropriate messages to strangers, giving a false impression of negative feelings or a lack of interest (p. 176). Yet, almost 20 years on, conscious controlled tone of voice is all but absent in AAC devices, and – perhaps more worryingly – in AAC research.

This oversight is in some way surprising, given that the individuality and identity of synthetic voices has become a focus of speech technology research and development. The inappropriateness of sharing an identical synthetic voice with other people is recognized, even ridiculed (Ridley, 2012). New technology is allowing custom synthetic voices to be created, taking into account regional accents, gender, and anatomy (e.g., Bunnell & Pennington, 2010) and this provides the foundation for voice banking and voice donation (e.g., Jreige, Patel, & Bunnell, 2009; Yamagishi, Veaux, King, & Renals, 2012). Portnuff (2006) asked us to reflect deeply on the ways in which people associate voice with identity. Of his own speech-generating device he said, “I guess I am beginning to identify with the voice myself, but I would still not hesitate to toss out the voice I use if I could get a more expressive one without sacrificing intelligibility” (p. 5).

In this paper, we encourage new approaches to tone of voice in AAC. We describe how an interdisciplinary project, Six Speaking Chairs, has provoked new perspectives and a social model of tone of voice. We anticipate challenges and opportunities for more expressive user interfaces, illustrated by the concept project Speech Hedge. We consider the role that new apparatus, such as the pilot project Tonetable, might play in enabling participatory research with people who use AAC and also seeding a research network around tone of voice. Finally, we speculate on how even more radical interactions might expand frontiers of AAC and disrupt speech technology as a whole.

## Provoking New Perspectives on Tone of Voice

Discussing tone of voice is surprisingly challenging. Often when laypeople and researchers alike talk about expressive speech, they talk about emotions. Artist Dan Keplinger, who was born with cerebral palsy and who has chosen not to use speech technology, states, “There is no way in hell a computer voice can express the emotion I have inside me” (Higginbotham, 2010, p. 62). Emotions are a part – one part – of expressiveness in AAC. There is also a preoccupation with emotions amongst speech technologists, and much of the work within this field has focused on synthesizing so-called emotional speech following the notion of the big six emotions: happiness, sadness, fear, surprise, anger, and disgust (Schröder, 2001). However, after recording and analyzing three years of daily conversation, Campbell (2005) found “the direct expression of emotion to be extremely rare” (p. 109). Campbell contends that when speech technologists talk about emotion in speech, what they really mean is that the current technologies are too text-based, and that increased expression of speaker attitude, affect, and discourse relationships is required (p. 109) – factors beyond basic emotions.

Alm and Newell (1996) argue the importance of “being an interesting conversation partner” in maintaining a full social presence (p. 181). Light (1989; Light & McNaughton, 2014) notes that sociorelational aspects of interaction in AAC have been more neglected than sociolinguistic aspects. She highlights the importance of relational skills including responsiveness to partners and the ability to put partners at ease (Light, 1989, p. 140), both of which are typically communicated using tone of voice. Portnuff (2006) identified challenges using AAC in an emotionally charged conversation: “I have gotten into hot water a few times saying something that I might have gotten away with by moderating my tone of voice” (p. 5). Keplinger's words (Higginbotham, 2010) suggest frustration with the limits of emotion and expressiveness, whereas Portnuff's speak of shortcomings in expressive nuance. Both are equally valid. Both need to be addressed in AAC research: We believe that emotion alone is too narrow a lens for examining tone of voice and how it functions within a social context.

### The Elusiveness of Tone of Voice

Higginbotham (2010) reflects deeply on expressiveness. He selects from Portnuff’s own words a narrower definition: “I want a question to sound like a question and an exclamation to sound like an exclamation” (p. 55), but leaves out sounding sensitive, sarcastic, sincere, suggestive or sexy. This reduction to punctuation, to an aspect that is included in text-to-speech, suggests how elusive a quality tone of voice is. Human speech has the ability to capture subtle shades of meaning beyond that conveyed by words and punctuation (Holmes & Holmes, 2001, p. 2). Yet for phoneticians “Intonation has traditionally been regarded as a problem” (Fox, 2000, p. 269) and even linguists are not able to describe all the nuances of meaning conveyed by what the listener perceives as intonation (Crystal, 1995, p. 248). Given how challenging tone of voice is to these experts, how then can a conversation about tone of voice be started amongst the rest of us?

### Embodying Ways of Thinking About Tone of Voice

In view of the elusiveness, the invisibility, and the intangibility of tone of voice, something was needed to seed the conversations that are so lacking. We adopted a methodology of deploying designed objects in early research in order to engage communities (Gaver, Dunne, & Pacenti, 1999). The role of these objects was not to propose actual designs for more expressive speech-generating devices, but to catalyze a deeper and more inclusive discussion about what we might want from them. They lie within a tradition of critical design, design practice employed in order to ask questions and provoke discussion rather than to (directly) solve problems or find answers (Dunne & Raby, 2001, p. 58).

**Figure 1.**
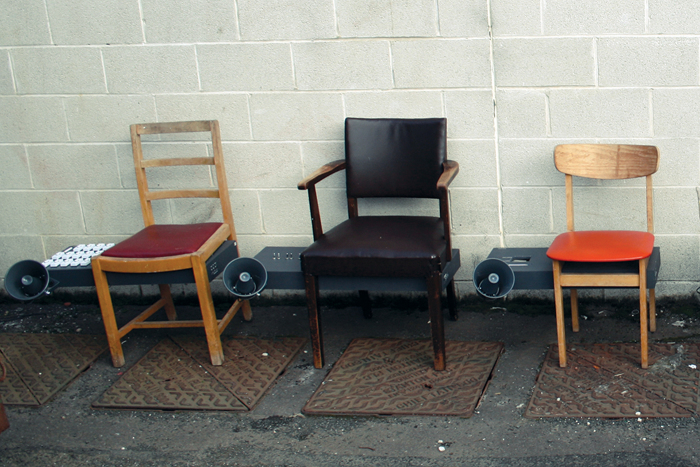
Three of the Six Speaking Chairs: Numbers 6, 3, and 4.

*Six Speaking Chairs project.* This project involved the creation of a collection of six chairs, three of which are shown in Figure 1. Each chair embodied a different way to think about tone of voice (Pullin & Cook, 2010). Inspiration was drawn across various academic and creative fields, ranging from sociolinguistics to the theater. The role of the chairs is to bring these diverse perspectives together to facilitate discussion: across disciplines and accessible to experts, non-experts, and people who use AAC alike. Just as the perspectives were pre-existing, found in other fields, so the chairs themselves were reclaimed from a furniture recycling project. The controls (dials, drumsticks, doorbells) were deliberately generic, familiar from people's everyday lives, to be used “without thought” (Fukasawa, 2007, pp. 6–7). The chairs served as visualizations. Iconic, old-fashioned metal horn loudspeakers on the front of each chair alluded to public address systems and embodied the potential for speech. But the chairs were also interactive. Each chair had a working user interface. A limited vocabulary allowed each interface to be prototyped quickly using a variety of speech technologies. The chairs were experience prototypes, wherein a person's experience is more important than the technology by which it is achieved (Buchenau & Fulton Suri, 2000).

It was also the intention to reverse the imbalance of current communication aids: Rather than being able to say many words in a very limited number of ways, these prototypes were capable of saying only four words. Sitting on each chair, using a control to the left of the seat, a participant could select between “*yes,*” “*no,*” “*really,*” and “*hello.*” “Yes” was chosen because while the word “yes” is usually affirmative in writing, in speech its meaning can be more complex, more layered, more sophisticated. With the right tone of voice, a person can either say *yes* to agree, to reassure, to bide for time, or even to undermine (without being so blunt as to actually say *no*). In the paper “Co-constructing meaning in conversations with an aphasic man,” Goodwin (1995) uses the notion of “. . . yes as a textured, non-binary answer” (p. 240). No one has yet described all of the nuances of meaning that can be conveyed by the single word, “yes,” even in an identical conversational context (Crystal, 1995, p. 248).

The word “really” is fascinating because it can be seen as a word with no meaning independent of the way in which it is said. The historian Paul Johnson quipped, “No man is truly English if he cannot say *really* seventeen different ways” (Pullin, 2009, p. 165). The word “hello” is powerful because, when uttered in a particular way, it can set the tone of the entire conversation that follows. If a person using AAC has less opportunity to frame a conversation in this manner, they are likely to relinquish much of this control to their conversational partner. Jakobson (1960) identified six verbal functions: expressive, directive, phatic, referential, poetic and metalinguistic. *Hello* said in different ways, can play any of these communicative roles (Pullin 2013, pp. 64–66). *Hello* is a greeting (the phatic function), that when uttered with sufficient portent, can inform of the seriousness of a situation (the referential function); when weighted with emotion can warn of the speaker's frame of mind (expressive function); when delivered with sufficient surprise or challenge can invite or demand an explanation (the directive function); and so on.

The range of conversational and social functions that can be carried by the tone of voice of the six chairs transcends the limited number of words involved.

Chair No. 1. The Exclaiming/Questioning Chair is a physical embodiment of the freedom of tone of voice offered by text-to-speech through punctuation. Its controls are three computer keys: a full stop (period), a question mark, and an exclamation mark. The expressive impoverishment of these punctuation marks is illuminated more clearly than when these three keys are hidden within a full alphanumeric keyboard on an AAC device.

Chair No. 2. The Happy/Sad Chair embodies emotional speech synthesis (Schröder, 2001). A radio dial is the main control with the names of radio stations having been replaced with emotional descriptors such as happy, sad, afraid, and surprised based on the Geneva Emotional Research Group mapping of emotions onto a circle (Scherer, 2005). The interaction is one of tuning into an emotional tone of voice.

Chair No. 3. The Offering/Seeking Chair adopts a more complex model of tone of voice (Campbell, 2005) that includes social-relational and contextual considerations. It has toggle switches for three parameters relating to self (including emotion), other (including relationship with conversational partner) and event (including conversational intent). Some of these settings would not change throughout a whole conversation.

Chair No. 4. The Rising/Falling Chair is a physical embodiment of the intonation diagrams that phoneticians have used for many years (Jones, 1936). The interaction is reversed, however: With this chair, the act of drawing the diagram creates the speech pattern. A touchscreen is embedded in the chair and drawing on it with a stylus shapes the intonation of the utterance: up and down for pitch and left to right for playing out the phonemes.

Chair No. 5. The Reassuring/Undermining Chair has a drum pad and drumsticks. Striking the drum pad triggers “*yes*” and “*no*”; more tentative tapping produces the paralinguistic utterances “*ye-yeah*” and “*uh-huh*” that can reassure or challenge a conversational partner without interrupting them. Drumsticks of different materials, including wood, felt, and beeswax, allude to different vocal qualities (Pullin & Cook, 2010, p. 42).

Chair No. 6. The Terse/Roaring Chair has 17 doorbells. Above each is a hand-written descriptor for various speaking styles including, appreciatively, brusquely, coaxing, coyly, explosively, and protesting. These are stage directions taken from a playwright's script (Shaw, 1916). Not being based on any theoretical model, these descriptors are more heterogeneous than those of any of the previous chairs.

**Figure 2.**
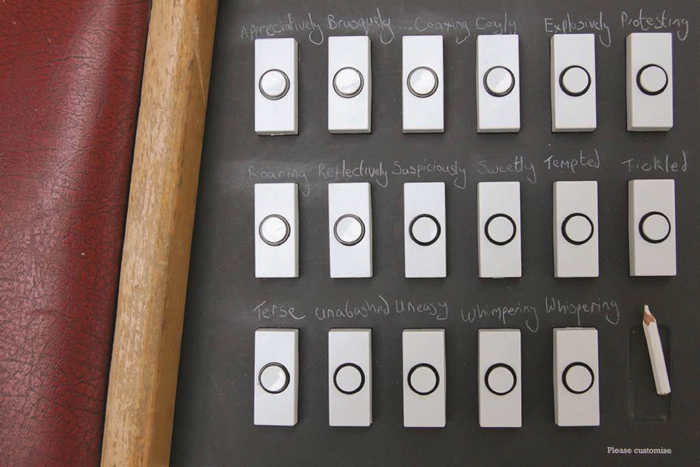
Detail from Chair Number 6: An invitation to contribute to the 17 Ways to Say Yes study.

**Figure 3.**
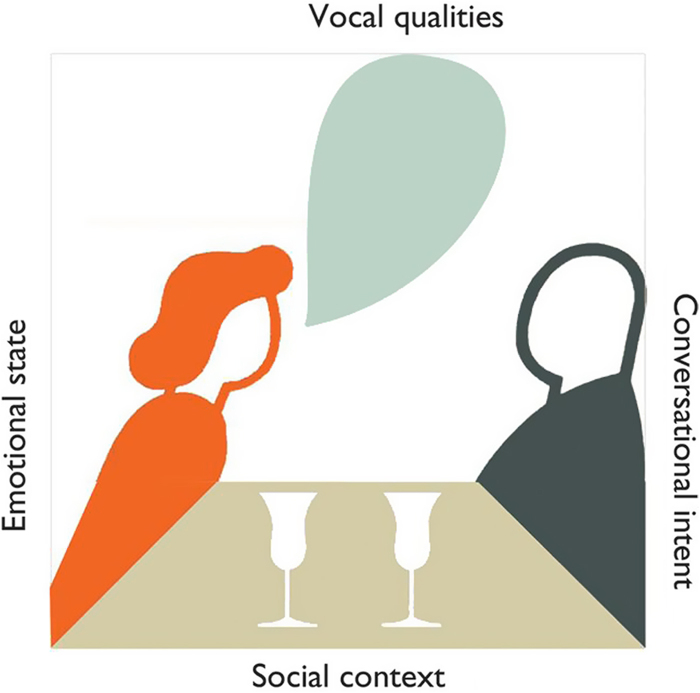
Four perspectives on tone of voice.

*17 Ways to Say Yes study.* In the bottom right-hand corner of Chair No. 6, after the last two doorbells marked “whimpering” and “whispering,” a short white pencil sits in a shallow trough, with the words “Please customise”, as shown in Figure 2. This invitation was used to frame a participatory exercise at the International Society for Augmentative and Alternative Communication 2008 conference in Montréal. First, a video was screened showing Chairs No. 1, 4, and 6 being deployed and demonstrated. Each participant was then asked to list alternative tones of voice that they would choose, if they were restricted to just 17 ways to say yes – while 17 would be a restriction in comparison to the speaking range of biological speech, for people using speech-generating devices, 17 options would represent an expansion of currently available options (Pullin & Cook, 2008). The responses from these 40 individuals familiar with AAC were collated. Even after combining equivalent terms such as anger, angrily and angry, this resulted in over 250 distinct descriptors. Some (e.g., angrily, apathetically, apprehensively) described the emotional state of the speaker, whereas many others (e.g., patronizingly, pleadingly, peacefully) carried additional connotations, such as the power relationship between the speaker and the listener. The 257 descriptors were then examined and sorted into categories. Four perspectives emerged: emotional state, conversational intent, social context (in terms of the speaker and listener’s relationship, status, or social setting), and vocal qualities (whether described directly or metaphorically). These perspectives are shown diagrammatically in Figure 3. The icons are taken from Gerd Arntz’s signs for the Isotype system (Neurath, 1936); like the conceptual frameworks and the chairs, these are yet more found objects.

**Figure 4.**
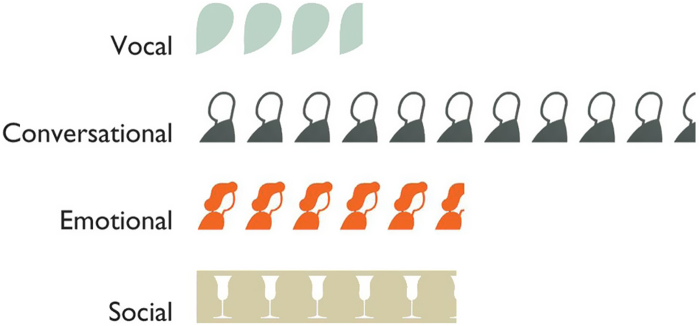
Mapping 257 responses against four perspectives. Each icon represents 10 responses.

When the 257 tones were mapped against these four perspectives, it was striking how few were classed as describing emotional states, as illustrated in Figure 4. Depending on how the mapping was carried out (whether or not weighting was given to the frequency of common descriptors such as angrily, enthusiastically or sarcastically) and by whom, emotional descriptors always accounted for less than half of the total responses; sometimes less than a tenth of the unique responses. Either way, it is a minority that we feel challenges the equivalence of expression and emotion, at least in the diverse ways in which people think about tone of voice.

The outcomes of the Six Speaking Chairs project were twofold: new engagement with the issue of tone of voice, and insights about the ways in which people already think about tone of voice. The role of the chairs themselves was pivotal but indirect. At the time of their creation it felt very important to us that they be interactive, and we even had plans (unrealized) for deploying them and assessing their relative effectiveness in controlled conversational contexts. Increasingly we understood that their initial purpose – to act as a catalyst – was valuable in itself. This represented a journey from a human-computer interaction methodology more traditionally employed within AAC to truly design-led research, as our confidence grew in contributing design sensibilities to this interdisciplinary field. It was the intriguing yet approachable presence of the Six Speaking Chairs that engaged people with this participatory research.

### Toward a Social Model of Tone of Voice

Echoing the change of perspective from a medical to a social model of disability, this paper proposes a move from an emotional to a social model of tone of voice. Like disability, tone of voice demands a perspective that is broader than the individual – one that encompasses not just the speaker's internal emotional state but also his or her cultural context, situational environment, social relationships, and individuality.

Clark (1996) argues that spoken interaction is often less about transferring information and more about social processes through which relationships are formed and maintained. Non-emotional aspects of tone of voice are thought to play a larger role in human interaction with respect to both frequency of use and functional impact, compared to emotional aspects. Future research is invited, perhaps applying descriptive or qualitative research methodologies, to validate or establish new perspectives of tone of voice.

## Anticipating New Interactions with Tone of Voice

Having argued that the very elusiveness of tone of voice has inhibited its adoption in AAC research, we acknowledge that another reason for its neglect has been technical. For many years, state-of-the-art speech synthesis has been based on concatenation (also known as unit selection) of samples of recorded speech. The clarity and credibility of this synthesized speech has been steadily improving but its tone of voice has been locked into a neutral reading style in which the speech corpora have typically been recorded. However, recent developments with parametric and hybrid approaches to speech synthesis (Bunnell & Pennington, 2010) offer the potential to unlock tone of voice. Statistical approaches, such as hidden Markov model-based speech synthesis, allow a credible voice to be generated from less recorded speech. This allows for more individuality of overall voice, in part because more diverse samples can be manipulated and still produce realistic neutrally read speech (Clark & King, 2006). Conversely, the same technology could be used to make the tone of voice of synthesized speech more flexible.

### Designing Interactions with Tone of Voice

In a chapter tantalizingly entitled “Interface Design for Speech Synthesis Systems,” Flach (2001) describes a user interface in which parameters such as fundamental frequency and spectral tilt can be specified at will in order to change the prosody of synthetic speech. The assumption is that the user is familiar and even fluent with these terms. Most speech technology is designed to be used by speech technologists, not by people who use AAC. Yet, Norman (2011), whose focus is on everyday interactions, argues that simple tools are often not up to the task; that technology needs to mirror the complexity and richness of our lives. The 17 Ways to Say Yes study illustrated how rich and complex tone of voice is. One challenge will be to create user interfaces for expressive speech synthesis that embrace this richness and complexity, whilst still being approachable, engaging, and not overly taxing to use.

**Figure 5.**
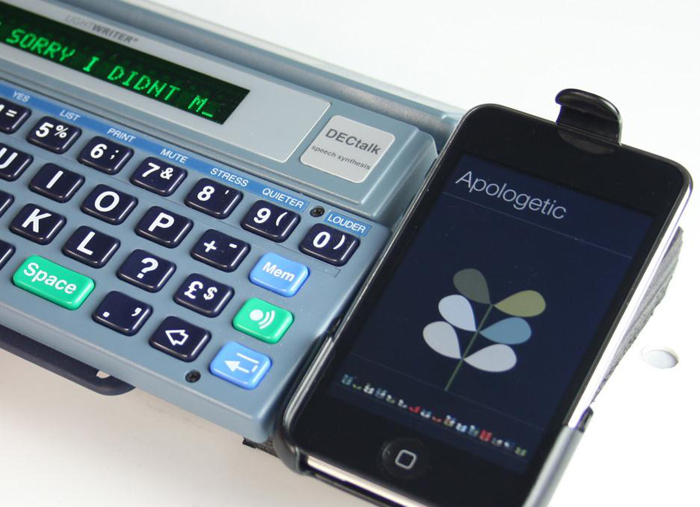
Speech Hedge interface for tone of voice, showing leaves, plants, and a hedge.

*Speech Hedge project.* This speculative design concept (Pullin, Cook, & McLeod, 2010; McLeod, 2010) proposed a radical new interaction. The concept extends the text-to-speech interface of a Toby Churchill Lightwriter SL35^™^^1^ (as an example of a speech- generating device) with a separate interface to control tone of voice. To visualize the concept, Figure 5 shows this extension running on a mobile phone; in practice the two interfaces could be combined, but their separation makes it clearer what has been added.

At the core of the Speech Hedge concept is the principle that complex tones of voice can be built up by combining simpler elements. These elements have descriptors covering the four perspectives on tone of voice found in the 17 Ways to Say Yes exercise: emotional (including pleased and energetic), social (including politely and formally), conversational (including questioning and confrontationally) and vocal (including breathily and rich voice). A distinct visual language is adopted, inspired by fabric design (Kiely, 2010), in order to convey a particular idiom: Each tone of voice is represented as a “plant” or seedling, made up of up to 8 of 16 alternative elemental tones, shown as different colored “leaves.” Once created, plants are organized into “hedges” of 16 plants, affording immediate access to a limited palette of tones of voice at any one time. People could collect and arrange their own palettes suitable for different contexts and circumstances: at a formal meeting, at a party, with a partner, and so on.

### Between Real-time and Pre-packaged

The dilemma for designing user interfaces is how to support richer tone of voice without further slowing conversation, which already compromises the ability to retain control of a conversation (Todman, 2000) or to even participate (Robillard, 1994). We proposed creating separate and complementary user interfaces: One, used in private, to assemble and design tones of voice for future use; the other, used in live conversation, to deploy the tones. The first can offer open-ended freedom to craft new tones of voice; the second constrains the choice to a limited palette when speaking.

This implies possibilities between or beyond Higginbotham’s (2010) two options for providing real-time expressive control, that is, by directly controlling prosodic dimensions, or by increasing choices between discrete pre-packaged prosodic variations (p. 65). In Speech Hedge, the in-conversation interaction is indeed a choice between variations, but rather than pre-packaged by the technology provider, they are created or collected by the individual through an interaction that would prove too demanding and distracting in live conversation. There are precedents for this in consumer mobile devices that offer constrained interaction in isolation but are configured and organized using a fuller user interface on a larger platform. An extreme example being Apple's iPod Shuffle^™^^2^, with no screen at all, relying on the iTunes^™^^2^ environment on another device with a screen. The challenge would be to integrate these elements into a coherent whole: Relating the micro scale of individual tones to the macro scale of myriad possibilities; unifying with a strong abstract model; applying a consistent design language; and, amongst this complexity, somehow instilling a feeling of space. Getting the interaction right is every bit as important and demanding as getting the technology working. Interaction design is the design discipline that plays this role professionally.

### Toward Open-ended Crafting of Tone of Voice

The model of leaves, plants and hedges is not the point of Speech Hedge; the accessibility of nuanced tone of voice is. This paper proposes that the AAC field adopts an aspiration of open-ended freedom to craft tone of voice. Rather than imagining future systems that offer an increment from the existing three punctuation options, we propose expanding future options by many orders of magnitude. By analogy, instead of moving from three colors to a dozen colors, we dream of pastels and acid colors, drabs and earth–tones, cool greys and warm greys, and their combination in rich palettes.

*A million ways to say yes study.* Speech Hedge has met with interest from AAC researchers, people who use AAC, and speech technologists. Most interestingly, it has opened up discussion about the way in which speech technology is developed, and by whom. As part of an audience response to Speech Hedge, participants were invited to draw metaphoric plants for different tones of voice, imagining how they might synthesize them by combining elemental tones. This exercise was introduced in terms of a million ways to say yes, because assembling a plant of between two and eight leaves, each leaf chosen from (the same) 16 alternatives, leads to over a million possible combinations.

Some responses illuminated the different perspectives on tone of voice already discussed. One respondent synthesized sarcastically by combining the tones for confrontationally and politely, while another combined the tones for emphasizing, loudly, and brusquely: the first reads as a top-down description of the role of sarcasm (and its inherent contradiction), the second reads as a bottom-up description of its sound. Other comparisons revealed a deeper issue: One respondent synthesized sarcastically by combining bored and emphasizing, while another assembled sarcastically from loudly, energetic, and emphasizing. This time, two different sounds were being described – the first reads as a deadpan delivery of sarcasm; the second, an ironically exaggerated delivery. We would argue that neither is more or less valid than the other.

### Culturally Specific Tone of Voice

What might be seen as a problem could, if embraced, catalyze a revolutionary approach to speech technology development. If we go beyond a gross distinction between anger, sadness, and happiness, then cultural influences will – and must – come into play. Light and McNaughton (2012) identify increased demands for culturally responsive AAC (p. 198). Responses such as *unimpressed* or *enthusiastically* are likely to be socially constructed. These tones may sound very different to different people – as might the words used to describe them.

A central feature of Speech Hedge is that once someone creates a tone of voice that they think might be useful, they subjectively label it after the event. In some ways this is a practical consideration: Because current speech technologies do not hold a comprehensive parametric model of tone of voice, it is this manual intervention that makes the concept technically feasible. But this practicality could define an ideal.

### Toward an Open Library of Tones of Voice

This paper proposes that people with disabilities, individually and collectively, might pioneer the proliferation of meaningful tones of voice for social purposes. This transcends the open source model already common in speech technology, given that this usually implies being open to a community of developers, rather than accessible to the people who use the technology in their everyday lives. There are precedents in other creative fields, including the Adobe Color CC^™^^4^ platform used by a community of graphic designers to share, exchange, and critique each other's color palettes (Adobe, 2015). Once again, the analogy may be apt: just as we need the cultural concept of color – not just the numerical values of wavelength or hue, saturation, and brightness – to discuss visual language, so do we need the higher level concept of tone of voice to explore the role of speech qualities in conversational interaction. We need to engage people with this new possibility of creating open community for tones of voice that includes new roles (Pullin, 2013, pp. 162–165). There should be space to encourage craftspeople to publish new tones, to enable speakers to browse new tones, to host a forum for the exchange of tones, and to seed a community in which diverse and hybrid roles are possible. Future research is needed to explore the idea of an open model, emphasizing the exchange of tones of voice rather than the actual means of creating them. Given its inherently emergent nature, something would need to be built and distributed in some way, and be used by a community (hopefully in some unexpected ways). This involves what is referred to in service design as a path to participation: The journey that each new participant takes, from awareness to adoption, advocacy, and appropriation.

## Framing New Research into Tone of Voice

Unfortunately for our community, expressive tone of voice has not been a priority for mainstream speech technology research. In most well-known commercial applications for speech technology – railway announcements, screen readers, automated telephone services, satellite navigation, and so on – expressiveness (especially when conceived of narrowly as emotional speech) can feel contrived. Thus, it may fall to the AAC community to drive investigations into expressive speech synthesis, given the unique issues presented by a lack of expressiveness.

### Investigating Tone of Voice

However interdisciplinary, the dominant culture in AAC research is scientific: AAC-RERCs (Rehabilitation Engineering Research Centers) hold State of the Science conferences. In offering key principles underlying research and practice in AAC, Blackstone, Williams, and Wilkins (2007) note that research must involve the active participation of people who rely on AAC (p. 193) and whose needs are best served by research that is guided by clearly articulated approaches to empirical observation and assessment (p. 194). It feels important, then, to bring the study of tone of voice into this research culture – a challenge in itself, given the elusiveness of tone of voice. At the same time, the active participation of people who use AAC demands that we do not rely on esoteric nomenclature to address the intangibility of tone of voice. We have to make it accessible. This presents a problem in anticipating and directing future capabilities for speech technology, which might be feasible but not yet executed. How is the case to be made for their development internally within AAC as much as externally to speech technology partners? How can we know what will be valuable within AAC? What is the evidence?

*Evaluating expressive speech study.* We can draw on some experience evaluating the use and effectiveness of more more expressive voices (Hennig, 2103; Hennig, Székely, Carson-Berndsen, & Chellali, 2012). A computer-based survey was developed in which sets of identical phrases were synthesized with three different tones of voice. Each set of synthetic utterances was embedded within a social scenario in which a theoretical AAC user expressed opinions and the participants took the role of outside observers. After each scenario, participants were given the written open-ended question, “How did the man sound?” Representative responses included: “sincere,” “keen,” “He sounded indifferent,” and “Insistent, rather than reassuring.” These responses were rated in terms of how effective the implied communication had been, knowing the communicative intent. For example, “I really believed that he meant what he said” was rated as effective, whereas “He didn't sound very keen” was rated as ineffective (given a particular social scenario). Perceived effectiveness was lowest when there was no variation in tone of voice.

### Embodying Investigation

Following the success of the Six Speaking Chairs in engaging an AAC research community, we believe that iconic research apparatus could play a role in giving physical form to an otherwise invisible and overlooked research issue. Making such investigation more accessible to the entire AAC community would also involve opening it up to researchers who don't have the technical skill to synthesize speech themselves and to people who use AAC, thereby catalyzing new and deeper lines of inquiry.

**Figure 6.**
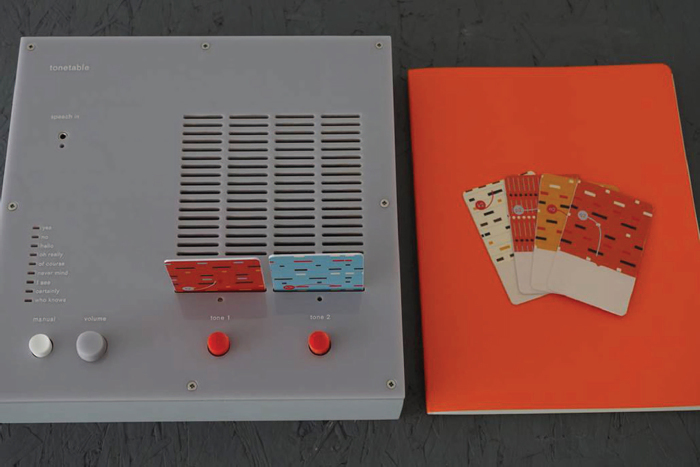
Tonetable experience prototype, showing box, cards, and notebook.

*Tonetable pilot project.* Still at the pilot study stage, shown in Figure 6, Tonetable is a first instance of such a research tool. It is a dedicated device that does not synthesize speech itself, but modifies the intonation (and eventually other qualities) of speech from any speech-generating device connected to it. The result is new tones of voice rather than new voices. The device is designed to be portable so that the ways in which tone of voice functions in specific social situations can be easily explored.

Each tone of voice is represented by a card in a deck of 22 cards. Participants can select a card and insert it into the Tonetable, which has two card reader slots to allow comparisons between two contrasting tones of voice. The cards do not describe the tones; they are differentiated by an abstract pattern and color palette, acting as a mnemonic to support a growing conversation between researcher and participant about each tone. Each card includes a blank area that can be written on, if participants wish to name or label a specific tone. Tonetable would also come with notebooks, pre-formatted with tables in which to record experiments and observations. The notes would be hand-written, but could later be photographed or scanned and then uploaded onto a communal research portal, where results would be shared and discussions hosted.

### Toward a Research Network Around Tone of Voice

The physical nature of the cards and Tonetable make it somewhat immutable. Even though new card sets could be issued, a limited collection of tones could allow a deeper study of what otherwise can be an overwhelmingly multidimensional quality, which could then also be shared between different researchers and research centers. The combination of a constrained yet distributed tool is what could illuminate the cultural aspects of tone of voice and so build a foundation for culturally responsive tone of voice. New investigations may be required and these, in turn, could enrich or challenge evolving theoretical constructs. For example, the notion of communication rate is increasingly conceived in terms of the establishment of shared meaning between conversational partners rather than narrowly in terms of words per minute (Blackstone et al., 2007, p. 194). It has long been established that a single word interjection, said in a particular way, can stand in for an entire sentence: “Yes” can mean either “Yes, of course it is so,” or “Yes, I understand that; please continue” (Jones, 1936, p. 257). We still need to be able to investigate this in the context of AAC. We would like to hear from any researchers interested in investigating any aspect of tone of voice, experimentally.

## Exploring New Frontiers of Tone of Voice

So far, all discussion has taken place within the paradigm of text-to-speech, which is now so prevalent that the term is used synonymously with speech synthesis. This is in contrast to earlier speech technologies, such as Wolfgang von Kempelen's mechanical speaking machine of 1791 (Pieraccini, 2012, p. 25) or Homer Dudley's analogue electronic VODER of 1939 (Pieraccini, 2012, p. 48), which had quite different user interfaces, and in which voice qualities and intonation (respectively) were physically manipulated as an integral part of the control of speech sounds.

### Playing With Tone of Voice

The paradigm of text-to-speech implies a relationship in which tone of voice is somehow a secondary optional layer. An analogy between text and tone of voice and melody and harmony is contained in Beukelman's (1989, p. 257) notion of “Things you just can't say with your right hand,” contrasting the roles of a pianist's right and left hand. Recent advances in speech technology have opened up the potential for more expressive prosody for its own sake, for example, in artistic installations and performance works (Astrinaki et al., 2012). Given that many people who use AAC also have impaired movement and dexterity, this kind of direct manipulation of speech sounds may not be appropriate. Some researchers have considered alternative ways to select tone of voices, for example, using facial expressions to control expressive speech synthesis (Székely, Ahmed, Hennig, Cabral, & Carson-Berndsen, 2014). Further approaches that involve labeling or describing tones of voice might demand metalinguistic skills that some people with complex communication needs either do not have or have not yet developed.

### Toward Toys for Infants to Play with Tone of Voice

Another possible frontier of the application of speech technology is whether and how infants with speech impairment might be given access to spoken tone of voice. In typical development, the use of tone of voice to communicate intent as well as emotion precedes speech and language (Crystal, 1969). There is also increasing awareness of the developmental role of AAC in language acquisition (Light & Drager, 2007). Other projects are exploring the role of tools in language acquisition: PhonicStick (Waller, Black, Martin, Pullin, & Abel, 2008) supports the development of phonological awareness by children with complex communication needs.

Toys to play with tone of voice might help to illuminate the role that this could play in language and social development of children with complex communication needs. Crystal (1975) acknowledges close links between intonation and affect but warns that the place of intonation in language acquisition is more complex (p. 150). Intonation itself is complex and involves grammatical, attitudinal, and social factors, with the relative importance of each varying considerably with the increasing complexity of the rest of a child's language (p. 153). This leads us to believe that a means to play with intonation and tone of voice may be valuable. The connotation of playing here is open-ended, perhaps without an outcome in mind, but learning and developing occur nonetheless.

### Toward a Revolution in AAC

Perhaps toys for playing with tone of voice would become stepping-stones to conventional text-to-speech devices; perhaps something could be learned from exploring AAC from the perspective of toys (Light, Drager, & Nemser, 2004). More radically still, perhaps new approaches to speech synthesis might emerge in which tone of voice is fundamental to the definition of speech, rather than a secondary consideration after linguistic content – one could think of this as “left handed” speech technology, to appropriate Beukelman's (1989, p. 257) analogy.

## Conclusion

The role of speech technology in AAC is one of the most challenging and most profound. In contrast to most speech technology applications, in AAC there is always a person at the center of the interaction. Despite critical differences, the current expectation in both fields is that AAC will inherit speech technology developed for other uses. This is the so-called trickle-down effect, whereby advances in military or mainstream technology find their way into specialist products for people with disabilities. Flow in the opposite direction is more radical (Pullin, 2009, p. xiii). Encouraging tone of voice in AAC research and practice could bring about a revolution in the field's relationship with mainstream speech technology: People who use AAC could become not just beneficiaries but also acknowledged pioneers. Portnuff (2006) closed his talk with the wish that each member of the AAC research community adopts a person or community with impaired speech, as mentor.

Spend time with us. Learn from us, and teach us. Share what you learn freely and openly with your colleagues. And hopefully, the rubber will occasionally meet the road, and your contributions will have a magnificent impact on someone's life. (p. 6)

Unfortunately, Portnuff is no longer with us. This is an invitation to other mentors, to participate with us and with others in the development of speech technologies that afford nuanced tone of voice.

## Notes

1. Lightwriter^®^ is a trademark of Toby Churchill, Ltd.

2. iPod Shuffle^®^ and iTunes^®^ are trademarks of Apple Inc.

3. Tango^®^ is a trademark of Dynavox

4. Adobe Color CC is an application from Adobe Systems Inc.
